# Primer to Voltage Imaging With ANNINE Dyes and Two-Photon Microscopy

**DOI:** 10.3389/fncel.2019.00321

**Published:** 2019-07-16

**Authors:** Bernd Kuhn, Christopher J. Roome

**Affiliations:** Okinawa Institute of Science and Technology Graduate University, Okinawa, Japan

**Keywords:** voltage-sensitive dye, VSD, voltage imaging, ANNINE, electrochromism, solvatochromism, membrane potential, two-photon

## Abstract

ANNINE-6 and ANNINE-6plus are voltage-sensitive dyes that when combined with two-photon microscopy are ideal for recording of neuronal voltages *in vivo*, in both bulk loaded tissue and the dendrites of single neurons. Here, we describe in detail but for a broad audience the voltage sensing mechanism of fast voltage-sensitive dyes, with a focus on ANNINE dyes, and how voltage imaging can be optimized with one-photon and two-photon excitation. Under optimized imaging conditions the key strengths of ANNINE dyes are their high sensitivity (0.5%/mV), neglectable bleaching and phototoxicity, a linear response to membrane potential, and a temporal resolution which is faster than the optical imaging devices currently used in neurobiology (order of nanoseconds). ANNINE dyes in combination with two-photon microscopy allow depth-resolved voltage imaging in bulk loaded tissue to study average membrane voltage oscillations and sensory responses. Alternatively, if ANNINE-6plus is applied internally, supra and sub threshold voltage changes can be recorded from dendrites of single neurons in awake animals. Interestingly, in our experience ANNINE-6plus labeling is impressively stable *in vivo*, such that voltage imaging from single Purkinje neuron dendrites can be performed for 2 weeks after a single electroporation of the neuron. Finally, to maximize their potential for neuroscience studies, voltage imaging with ANNINE dyes and two-photon microscopy can be combined with electrophysiological recording, calcium imaging, and/or pharmacology, even in awake animals.

## Introduction

ANNINE-6 and ANNINE-6plus are voltage-sensitive dyes successfully implemented for *in vivo* imaging with two-photon microscopy in bulk loaded tissue (Kuhn et al., 2008) as well as in dendrites of single neurons (Roome and Kuhn, 2018). ANNINE dyes (Hübener et al., 2003; Kuhn and Fromherz, 2003; Fromherz et al., 2008) were developed and designed based on elaborate physical chemistry studies and theoretical models in the laboratory of Peter Fromherz (Ephardt and Fromherz, 1991, 1993; Fromherz and Heilemann, 1992; Fromherz, 1995; Röcker et al., 1996). ANNINE-6 was first synthesized in 1996, but it took several years of optimizations until effective protocols for voltage imaging (Kuhn et al., 2004) and labeling (Kuhn et al., 2008; Pages et al., 2011; Roome and Kuhn, 2014, 2018) were established. Here, we describe the design and voltage-sensing mechanism of fast voltage-sensitive dyes, specifically of ANNINE dyes, and how to optimize voltage imaging. In a second publication, we supply protocols for different *in vitro* and *in vivo* applications (Kuhn and Roome, 2019).

## Design and Membrane Labeling of Fast Voltage-Sensitive Dyes

The cytosol of all living cells has a negative electrical potential in relation to the extracellular space (which is at ground). The cytosol is conductive due to ions and it is electrically isolated from the extracellular space by a lipid bilayer; the cell membrane. Therefore, the interior of a cell is typically equipotential and the negative electrical potential drops across the membrane. For neurons, the potential across the membrane at rest is around -60 mV, and a typical action potential rapidly reverses the membrane potential to +40 mV. A change of 100 mV seems small, but since this membrane potential change occurs across a narrow cellular membrane, approximately 5 nm in width, the electric field change across the membrane during an action potential is dramatic; ∼200,000 V/cm (for comparison: the dielectric strength of dry air is 30,000 V/cm).

**Figure 1:**
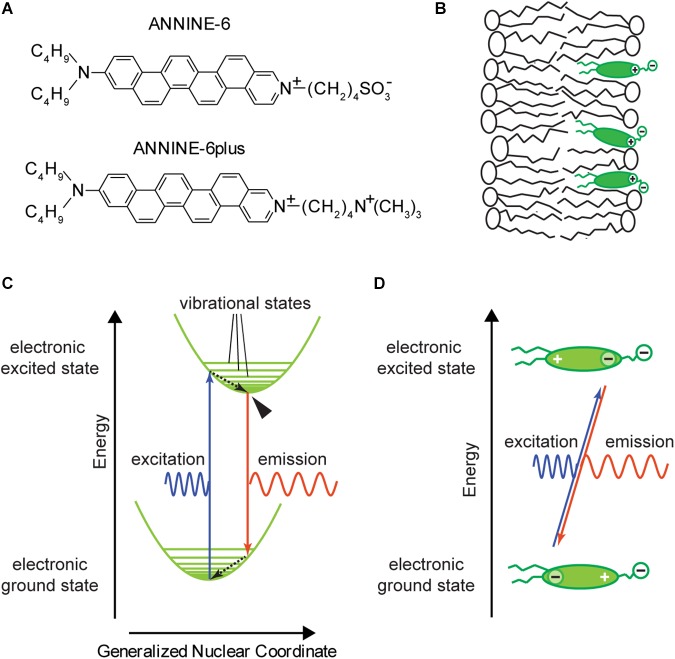
ANNINE-6 and ANNINE-6plus; basic properties. **(A)** Structure of ANNINE-6 and ANNINE-6plus. Both have a charged and therefore hydrophilic head group (right) and an uncharged hydrophobic tail (left). ANNINE-6plus is more water soluble than ANNINE-6 due to two positive charges compared to a positive and a negative charge. **(B)** Sketch of a voltage-sensitive dye molecules bound to a lipid membrane. The hydrophilic head groups align with the lipid head groups. The hydrophobic tail and chromophore are surrounded by hydrophobic hydrocarbon chains of the lipid molecules. **(C)** Franck–Condon energy diagram showing electronic ground and excited states over the generalized nuclear coordinate. The Franck–Condon energy diagram indicates that the electron transitions of the excitation and emission process are much faster than the adjustment of the new chromophore conformation, indicated by the generalized nuclear coordinate. After excitation and emission, the molecule vibrates around the new equilibrium position until it relaxes within picoseconds to the vibrational ground state. The vibrational ground state of the electronic excited state from which fluorescence occurs is marked by an arrowhead. Dashed arrows indicate radiationless transitions. **(D)** Charge shift within a voltage-sensitive dye during the absorption and emission process. During the absorption process, the center of charge of the outermost electron of the chromophore shifts from the aniline toward pyridinium. This shift occurs due to the asymmetry of the chromophore. For ANNINE-6 this charge shift is 0.81 nm (Kuhn and Fromherz, 2003). For comparison, the length of the chromophore is about 1.48 nm. During the emission process the electron moves back from pyridinium to aniline.

Such potentials and potential changes are typically measured using an electrode placed in the cytosol and compared against an extracellular bath electrode. However, if we want to design an optical probe that senses the potential locally, it must be physically located inside the membrane or, at least, within the Debye length of the membrane (i.e., the length constant of the electrostatic field generated by charges close to the membrane surface that attenuates into the surrounding electrolyte). A molecular probe that randomly diffuses within the conducting cytosol will not be exposed to any significant potential changes, as any potential difference will quickly shift charges until the cytosol is equipotential again. Therefore, fast voltage-sensitive dyes (Loew et al., 1978; Loew and Simpson, 1981; Fluhler et al., 1985), like ANNINE-6 (Hübener et al., 2003) or ANNINE-6plus (Fromherz et al., 2008) (Figure 1A), are designed to be incorporated into the cell membrane (Figure 1B). To do this, they necessarily comprise of both hydrophobic (from the Greek words hydro = water and phobos = fear) and hydrophilic (from the Greek word philos = love) components. At one end of the dye molecule there are two hydrophobic hydrocarbon chains serving as membrane anchors. At the opposite end there is a positively charged (and therefore hydrophilic) pyridinium group and a positively (or negatively) charged, hydrophilic head group. In between there is a mostly hydrophobic chromophore consisting of aniline and the anellated benzene rings. Such molecules with both hydrophobic and hydrophilic parts are called amphiphilic (from the Greek word amphi = both). The hydrophilic pyridinium and head groups align with membrane lipid head groups while the hydrophobic part of the dye ends up in the hydrophobic core of the membrane. The idea is that the linear design of ANNINE dyes promotes parallel alignment with the lipid molecules of the membrane so that the long axis of the dye is oriented parallel to the electric field across the membrane. However, it is unlikely that this perfect orientation is achieved.

Integration of amphiphilic dyes into the membrane is based on the hydrophobic effect. The hydrophobic effect is predominantly entropy-driven (entropy is a measure for disorder and maximizes at equilibrium): Neighboring non-polar molecules and polar water molecules cannot form hydrogen bonds. This reduces the number of possible hydrogen bonds of polar water molecule with their neighbors. Fewer choices brings more order, or less entropy. As the entropy of a system aims for maximization, the surface between hydrophobic and hydrophilic substances will be minimized corresponding to the largest number of possible choices and highest disorder. Therefore, oily, non-polar substances and water separate into two phases, and an amphiphilic voltage-sensitive dye in an aqueous environment will label a lipid membrane as soon as diffusion brings them in contact. So, in a first approximation, the binding process is entropy driven and binding forces can be neglected. For a detailed explanation of the hydrophobic effect see, for example (Dill and Bromberg, 2011). In general, the membrane binding strength, indicated by the free energy of binding, increases linearly with the length of the hydrophobic alkyl tail (Fromherz and Röcker, 1994; Hinner et al., 2009) and can therefore be easily manipulated.

## Excitation and Fluorescence of Fast Voltage-Sensitive Dyes

The excitation and fluorescence of voltage-sensitive dyes follow the same mechanisms as those of other fluorescent dyes but with some specifically optimized features. For a general introduction to the field of molecular fluorescence see Valeur and Berberan-Santos (2013) and Jameson (2014). In short, molecular chromophores consist mainly of carbon atoms joined by conjugated single-double bonds. In the structural formula, this is indicated by alternating single-double bonds between carbon atoms (Figure 1A). While bonding electrons in a covalent single bond are localized in an orbital between the two atoms, the electrons of a conjugated single-double bond system are delocalized in an orbital spanning the full conjugated system. Delocalized electrons are weakly bound so that the energy of a photon in the visible wavelength range is enough to bring the electron to an excited state. If a photon interacts with a molecule with conjugated single-double bonds, there is a probability that the photon will be absorbed. If so, the photon will disappear, and the energy and quantum numbers of the photon, which must be preserved, will be transferred to the molecule. To go back to the ground state, the molecule can emit a photon. Again, the overall energy and the quantum numbers of the molecule-photon system must be preserved.

**Figure 2:**
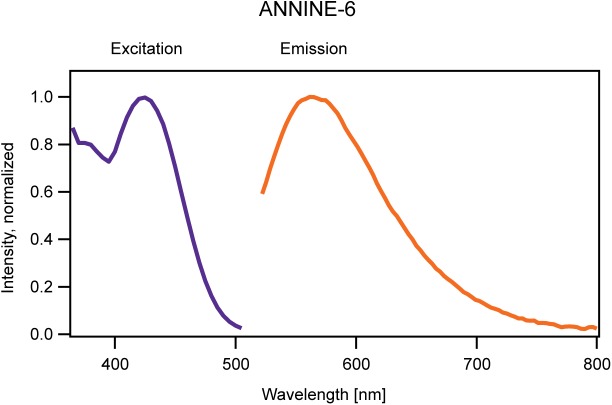
One-dimensional excitation (blue) and emission (red) spectrum of ANNINE-6 measured in a neuronal membrane (Kuhn and Fromherz, 2003). The intensity of the excitation spectrum was measured at the peak of the emission spectrum (565 nm). The excitation spectrum acts here as a proxy for the absorption spectrum mentioned in the text. To measure the emission spectrum ANNINE-6 was excited at the peak of the excitation spectrum (420 nm). Both spectra are normalized.

To describe the absorption and emission process, a molecular model applying the Franck-Condon principle is used. The Franck–Condon principle states that electronic transitions in a molecule are very fast in comparison to changes of bonding angles and bonding distances between the nuclei of the molecule. As electronic and nuclear transitions occur on different time scales, they can be separated into different processes. Figure 1C show the Franck–Condon energy diagram of a voltage-sensitive dye where molecular energy levels are plotted over a spatial axis which represents a generalized nuclear coordinate. Electronic transitions occur on a timescale of femtoseconds (10^-15^ s) and are indicated as vertical arrows corresponding to a change in electron energy, but no change in the generalized nuclear coordinate. The transition between two energy levels occurs with a specific probability. The different probabilities of transition between different levels are reflected in the shape of the spectrum (Figure 2). For example, the maximum intensity of the spectrum corresponds to the highest transition probability. After excitation, the molecule vibrates around the new equilibrium state and relaxes to the vibrational ground state on a time scale of typically picoseconds (10^-12^ s). During this relaxation process the molecule passes through a multitude of vibrational levels thereby dissipating the vibrational energy to the local neighborhood of the molecule as heat. The change of the generalized nuclear coordinate of the molecule is interpreted as a change of the size or shape of the molecule. This change will affect the location of the center of charge.

The vibrational ground state of the electronic excited state has a typical lifetime of nanoseconds (10^-9^ s). If a photon is emitted, the molecule arrives at a vibrationally excited, electronic ground state. As the relaxation time is much shorter than the lifetime of the excited state almost all photons are emitted from the vibrational ground state of the excited state (arrowhead in Figure 1C). Again, this transition can result in different vibrational levels with different probabilities within the electronic ground state, reflected in the shape of the emission spectrum (Figure 2).

During the relaxation processes following the absorption and emission of a photon, energy is converted into heat. Therefore, the emission spectrum is shifted to lower energy (or longer wavelength) compared to the absorption spectrum. The wavelength difference between excitation and emission peak is called Stokes shift, named after George G. Stokes (1819–1903) who described this wavelength shift for the first time (Stokes, 1952). Electrochromic voltage-sensitive dyes typically have large Stokes shifts. For example, ANNINE-6 and ANNINE-6plus show a Stokes shift of about 145 nm in a cell membrane as can be seen in Figure 2 (excitation maximum at 420 nm, emission maximum at 565 nm). For comparison, the enhanced Green Fluorescent Protein (eGFP) has a Stokes shift of about 25 nm. So, if an electrochromic voltage-sensitive dye is excited at the absorption maximum a significant fraction of the absorbed energy is converted into heat (in the case of ANNINE-6 and ANNINE-6plus, more than 20%), and heat generation should be minimized to avoid damage to the local environment (lipid molecules and membrane proteins).

The emission of a photon after absorption and relaxation is one process of deactivation. However, other processes can also occur, which are not shown in Figure 1C. For example, it is possible that the energy dissipates without emission of a photon. Another possible process is intersystem crossing, which causes phototoxicity: Typically, electrons in molecular orbitals are paired (if they are not paired, the molecule is very reactive). Every electron has a quantum mechanical property called spin, which is an intrinsic form of angular momentum. The spin of an electron Ŝ = 1/2. In molecular orbitals occupied by two electrons, the spins are oriented anti-parallel and therefore the overall spin of the system Ŝ = 0. This state is called singlet state because it is associated with only a single energy level. Molecules generally reside in the singlet state. However, molecular oxygen is an exception to the rule. In its ground state the spin of both outermost electrons is parallel and adds up to Ŝ = 1. Such a spin system can be split up by an external magnetic field into three energy levels. Therefore, it is called a triplet state. Now the following can happen: If a dye molecule is in an excited singlet spin state, and a triplet state oxygen molecule is nearby, the molecule can cross over into the triplet state while the oxygen molecule crosses over into the singlet state (Kalyanaraman et al., 1987). Such a process is called intersystem crossing. Singlet oxygen is a very reactive molecule which will quickly oxidize a neighboring molecule and thereby destroy its functionality. This process is especially harmful if membrane proteins are damaged. The dye molecule will remain in the electronic excited triplet state until it dissipates the energy. This can happen either by emission of a photon, a process called phosphorescence, or by a non-radiative process. In both cases, a neighboring molecule is necessary for returning from the electronic-excited triplet state (Ŝ = 1) to the electronic ground and singlet state (Ŝ = 0). The reason for this is that the transition from the triplet state to the electronic ground and singlet state is a quantum mechanically forbidden transition. A second molecule is necessary to conserve the quantum numbers during the transition.

Another important design characteristic of the chromophore of fast voltage-sensitive dyes is the asymmetry of the chromophore (Loew et al., 1978): One nitrogen atom is integrated into a carbon ring (pyridinium) while a second nitrogen atom is attached to the carbon ring (aniline). These molecules are classified as hemicyanine dyes because their chromophore includes nitrogen atoms, but they lack the symmetry of regular cyanine dyes. Most importantly, in the electronic ground state, this asymmetry results in an asymmetric delocalized electron system with the center of a positive charge at the pyridinium group and a delocalized electron at the aniline group (Figure 1D, bottom). During the absorption process, the center of positive charge shifts from pyridinium toward aniline, and during the emission process it shifts back to pyridinium. Or, if we consider the movement of an electron, the delocalized electron shifts from aniline toward pyridinium, and during the emission process back to aniline (Figure 1D, top). Therefore, this type of voltage-sensitive dye is also called a charge-shift probe.

## Mechanism of Voltage Sensitivity of Fast Voltage-Sensitive Dyes

**Figure 3:**
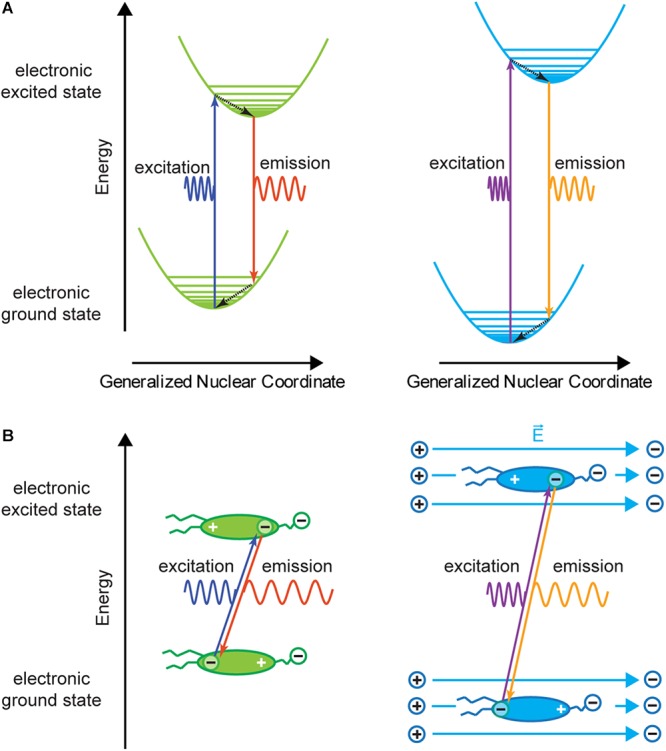
Franck–Condon diagram and charge shift without (left) and with (right) external electric field. **(A)** The energy levels in the presence of an external electric field are shifted compared to the energy levels without external electric field. This results in a change in both excitation and emission wavelengths for a given transition. Therefore, for a photon with a given wavelength the absorption probability will be different with and without an external electric field. In general, the energy shift due to the electric field is small compared to the transition energy. **(B)** Charge shift within a voltage-sensitive dye during the absorption and emission process. If the dye molecule is in an external electric field $\vec{E}$, charges within the molecule are slightly shifted (increasing or decreasing the polarization of the molecule) and have different potential energy (**A,B**, right). Therefore, transition energies are shifted compared to transitions without external electric field. In this example, the molecule is more polarized, and excitation and emission spectra will be shifted to higher energy. This corresponds to a spectral blue shift. If the electric field has the opposite direction, a red shift of absorption and emission spectra is expected.

If the above described charge shift occurs in an external electric field, in this case the electric field over the cell membrane, more or less energy is required for excitation and released during emission because work is done against the external electric field or work is done by the external electric field, respectively (Figure 3). This energy change results in an equal, or symmetric spectral shift of the excitation and the emission spectra. For example, if the negative center of charge of the delocalized electron must be moved in the same direction as the electric field (electric field lines emanate from a positive charge toward a negative charge) more excitation energy is needed than without the external electric field. When the electron drops to a lower energy level, it moves against the external electric field and gains energy so that the emission is also shifted toward higher energy. A voltage-sensitive dye that shows a spectral shift for both excitation and emission is called an electrochromic dye, and if the spectral shift is the same for excitation and emission on an energy-proportional scale it is called a pure electrochromic dye.

From a quantum mechanics point of view, the external electric field will cause splitting and shifting of energy levels of the chromophore. This effect is called molecular Stark effect (Loew et al., 1978; Kuhn and Fromherz, 2003), named after Johannes Stark (1874 – 1957) who discovered this effect in the excited state of hydrogen atoms (Stark, 1914). As the external electric field is small in comparison to intra-molecular electric fields, modulation of the orbital is small and can be approximated as being linear.

Interestingly, conversion of the electric field into the modulation of the optical signal occurs during both the absorption and the emission process. Both occur on a time scale of 10^-15^ s. This is the time it takes a photon, whose length equals basically its wavelength, to pass by a molecule with speed of light. However, the readout is delayed by the lifetime of the excited state which is in the nanosecond range. In the case of ANNINE-6plus, the fluorescence lifetime is 6.2 ± 0.1 ns (Roome and Kuhn, 2018). This high temporal resolution can be used to measure, for example, break-through voltages of membranes with a temporal resolution of 5 ns by ultrafast laser pulse excitation (Frey et al., 2006).

In general, the theoretical limit of voltage imaging with ANNINE dyes is even faster. It can be shortened to the duration of the absorption process (<10^-15^ s). This can be achieved by excitation at the red spectral edge of the absorption spectrum and integration of the full emission spectrum (see Optimizing Voltage Imaging). Thereby, voltage imaging becomes independent of the shift of the emission spectrum and (after deconvolution with the time-resolved fluorescence response) the optical readout reflects the applied voltage during the absorption process only.

**Figure 4:**
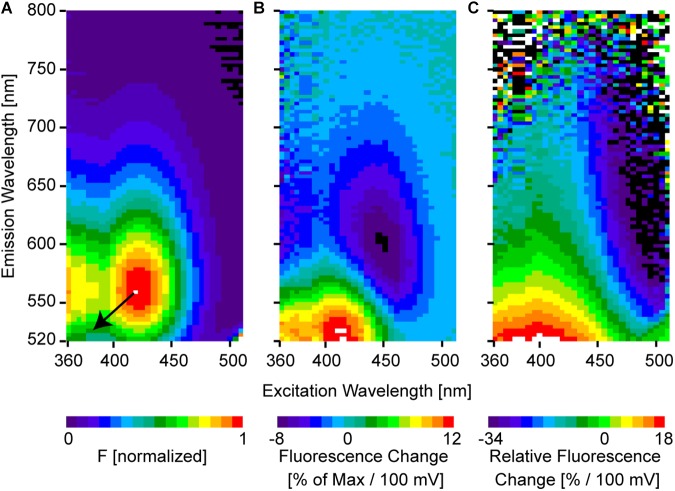
Two-dimensional spectra of ANNINE-6. **(A)** Two-dimensional fluorescence spectrum shows the intensity for every excitation-emission wavelength pair. The spectrum was measured from an ANNINE-6-labeled leech neuron. The spectrum is normalized to the maximum. Two such fluorescence spectra were measured, one at rest and another at depolarized membrane potential, with 100 mV potential difference. These two spectra were used to calculate **(B)** the two-dimensional fluorescence change $\frac{\text{Δ}F}{100mV}$ and **(C)** the two-dimensional relative fluorescence change $\frac{\text{Δ}F}{F}\frac{1}{100mV}$. The color code indicates the % change per 100 mV. The resulting pattern can be simply explained by a pure diagonal shift of the two-dimensional spectrum indicated by the black arrow in **(A)** (arrow indicates the direction of the shift, but shift is not indicated to scale; the real shift is only about 3–4 nm). An equal energy shift of both, the excitation and the emission spectrum, in response to a change of the external electric field indicates a pure electrochromic mechanism of voltage-sensitivity of ANNINE-6 and ANNINE-6plus (Kuhn and Fromherz, 2003; Fromherz et al., 2008).

The electrochromic effect can be best seen in two-dimensional fluorescence spectra in which the fluorescence intensity is plotted for every excitation wavelength λ_Ex_ and emission wavelength λ_Em_ pair (Figure 4A). Here, we use for convenience a wavelength scale which is proportional to 1/energy. In general, as the spectral shift is proportional to energy it would be better to use an energy-proportional scale, such as a wavenumber scale (1/cm) (Kuhn and Fromherz, 2003), which is widely used in physical chemistry, but rarely in the imaging literature.

For normalization, the two-dimensional fluorescence spectrum *F*(λ_*Ex*_, λ_*Em*_) is divided by the global maximum MAX(F(λ_*Ex*_, λ_*Em*_)) (Figure 4A):

$$F({\text{λ}}_{Ex},\;{\text{λ}}_{Em})\;=\;\frac{F_{rest}({\text{λ}}_{Ex},\;{\text{λ}}_{Em})}{MAX(F_{rest}({\text{λ}}_{Ex},\;{\text{λ}}_{Em}))}$$

The spectrum measured in the membrane of a cell at resting potential is F_*rest*_. After measuring F_*rest*_, the intracellular potential is changed by 100 mV, and a second spectrum is measured (F_*depolarized*_). Now, the spectral changes induced by the voltage can be analyzed. The two spectra look almost the same at first sight. For example, the largest spectral shift recorded so far (ANNINE-6) for a 100 mV membrane voltage change is only about 3 nm in excitation and 4 nm in emission (spectral shift of excitation and emission in wavenumbers: 160 cm^-1^). This spectral shift corresponds to a charge shift of 0.81 nm within the chromophore (Kuhn and Fromherz, 2003) (for comparison, the chromophore of ANNINE-6 has a length of 1.48 nm). However, when calculating the fluorescence change Δ*F*(λ_Ex_, λ_Em_), the shift becomes visible (Figure 4B).

$$\text{Δ}F({\text{λ}}_{Ex},\;{\text{λ}}_{Em})[\%/100\;mV]\;=\;\frac{F_{depolarized\;}({\text{λ}}_{Ex},\;{\text{λ}}_{Em})\;-\;F_{rest}({\text{λ}}_{Ex},\;{\text{λ}}_{Em})}{MAX(F_{rest}({\text{λ}}_{Ex},\;{\text{λ}}_{Em}))}100\%$$

As expected from a pure shift of the excitation and emission spectra, a region of positive and negative change is located symmetrically to the diagonal through the global maximum of the 2D fluorescence spectrum (red and blue extremes in Figure 4B). The unit of fluorescence change is % change relative to the maximal intensity of the two-dimensional spectrum for a voltage change of 100 mV. Importantly, there is no fluorescence change at the global maximum of the 2D fluorescence spectrum and along the diagonal through the global maximum.

Even more interesting than the fluorescence change is the relative fluorescence change $\frac{\text{Δ}F}{F}({\text{λ}}_{Ex},\;{\text{λ}}_{Em})$ (Figure 4C).

$$\frac{\text{Δ}F}{F}({\text{λ}}_{Ex},\;{\text{λ}}_{Em})[\%/100\;mV]\;=\;\frac{F_{depolarized\;}({\text{λ}}_{Ex},\;{\text{λ}}_{Em})\;-\;F_{rest}({\text{λ}}_{Ex},\;{\text{λ}}_{Em})}{F_{rest}({\text{λ}}_{Ex},\;{\text{λ}}_{Em})}100\%$$

The relative fluorescence change indicates the % fluorescence change for a voltage change of 100 mV at a given excitation and emission wavelength pair. In general, relative fluorescence change represents information that can be gained by detecting photons when exciting at λ_Ex_ and detecting at λ_Em_. Additionally, it is normalized to the number of photons, so the % change corresponds to information gain per detected photon. Therefore, relative fluorescence change should be optimized for functional imaging as a crucial factor in the signal-to-noise ratio (see below). Importantly, the largest (positive and negative) relative fluorescence change, which contains the most information about a voltage change, is found at the spectral edges of the 2D spectrum. For example, the intensity at an excitation wavelength of 500 nm and an emission wavelength of 560 nm is less than 10% of the global maximum (Figure 4A), but the sensitivity is -34%/100 mV (Figure 4C). But, at the absorption and emission peak, 420 and 560 nm, respectively, the sensitivity is 0%/100 mV.

It is important that the voltage-sensitive dye molecules are only on one side of the membrane. If voltage-sensitive dye molecules label both leaflets of the membrane bilayer, the spectral shift in response to the membrane voltage change occurs in opposite directions and therefore the relative fluorescence change will be inverted and will cancel out. For example, for excitation at 500 nm and labeling the outer or inner leaflet of the cell membrane, a negative or positive fluorescence change, respectively, is expected in the range of 550 to 750 nm (Figure 4B). Consequently, if dye molecules are equally distributed on both sides of the membrane, the voltage signals of the dye molecules on the two opposing sides of the membrane will cancel each other out. Labeling of both leaflets of the cell membrane can have several reasons: (1) It can occur if dye molecules flip from one membrane leaflet to the other. The flipping probability depends on the hydrophilic and hydrophobic properties of the dye. (2) An inappropriate labeling technique might cause labeling of both membrane leaflets. (3) If single cells are filled with voltage-sensitive dye and, by accident, dye is spilled extracellularly. And (4) due to a net charge of the dye headgroup, dye molecules might flip. For example, externally applied VSD molecules with a positive net charge are attracted by the negative potential inside the cell and might flip to the inside membrane leaflet. Therefore, the voltage signal might even invert (Tsau et al., 1996).

In general, this spectral shift, resulting in positive and negative sensitivity at two diagonal opposing slopes and no change at the global maximum of the 2D excitation/emission spectrum, is different from most other molecular probes. For example, most widely used calcium indicators, like G-CaMP (indicator family developed by J. Nakai) (Ohkura et al., 2012), GCaMP (indicator family of the GENIE Project) (Chen et al., 2013), or fluo dyes (Tsien, 1999), show the same relative fluorescence change for any combination of excitation and emission wavelengths for a given calcium change. Their mechanism is based on a change of absorption cross-section or quantum efficiency. In other cases, the equilibrium between two conformations or binding states of the probe is altered, and the ratio between two emission bands changes, as for the calcium sensitive dye indo-1, for example (Tsien, 1999). Therefore, the intensity or the equilibrium between two distinct excitation or emission spectra changes, but there is typically no spectral shift as in charge-shift probes by electrochromism and solvatochromism (but there are some exceptions, see Tsien, 1999).

A change of the optical voltage signal, as a result of a change of the excitation wavelength, can be shown in an experiment as a clear indicator that a voltage signal was recorded and that the voltage sensing mechanism holds.

## Two-Photon Excitation of Voltage-Sensitive Dyes

**Figure 5:**
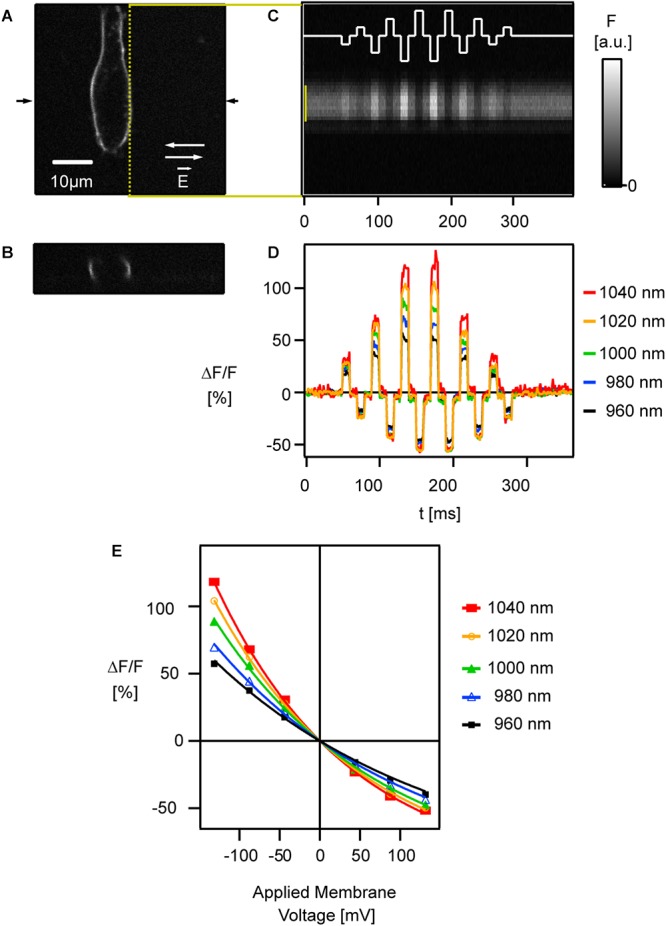
Relative fluorescence change of ANNINE-6 increases toward the red spectral edge of two-photon absorption. **(A)** A HEK293 cell labeled with ANNINE-6 is shown in the xy plane and **(B)** the xz plane at the location indicated by arrows in **(A)**. A two-photon line scan was taken along the membrane [yellow dotted line in **(A)**]. **(C)** External electric fields $\vec{E}$ with different amplitudes and direction [white trace, switching directions indicated in **(A)** by white arrows] were applied while scanning along the membrane. The spatio-temporal map shows bright and dark stripes correlating with the applied external electric fields. Intensity gray scale is given in arbitrary units [a.u.]. **(D)** Line scan bands [indicated by yellow bar in **(C)**] were spatially averaged to show relative fluorescence changes corresponding to six different (three positive, three negative) membrane voltage changes. Relative fluorescence changes increase with increasing excitation wavelength, i.e., closer to the red spectral edge of the absorption spectrum. Care was taken that laser polarization was parallel to the main axis of voltage-sensitive dye molecules and therefore perpendicular to the scanned membrane. **(E)** The relative fluorescence change [symbols indicate average voltage response during an electric field pulse at a given wavelength seen in **(D)**] increases linearly in the typical voltage range for biological depolarization and hyperpolarization (±80 mV from resting potential). Only for high voltage changes (>150 mV) the response deviates slightly from being linear. The sensitivity increases with higher excitation wavelength. Modified with permission from Elsevier (Kuhn et al., 2004).

The two-photon absorption probability depends on the change of the molecular dipole moment during the absorption process (Theer et al., 2005; Pawlicki et al., 2009). As the name implies, charge-shift dyes show a large change in polarization during the absorption process and are therefore well suited for two-photon excitation (Figure 5A). As with the one-photon process, two-photon absorption depends on the angle between the polarization of the excitation light and the molecular dipole moment, as the oscillations of the light’s electric field interact with the electric dipole of the molecule. This is important because voltage-sensitive dye molecules are well aligned in membranes. If the polarization of the excitation light and the dipole moment of the dye are perpendicular to each other, almost no fluorescence will be observed, as can be seen in the cross section of an HEK293 cell (Figure 5B) where the adhesion zone and the opposing membrane are invisible while the rim shows bright fluorescence. This effect can be observed with one-photon excitation, but it affects two-photon excitation quadratically because two photons must be oriented in the direction of the molecular dipole moment. Therefore, the polarization of the excitation laser light used for two-photon voltage imaging needs to be carefully considered.

As two photons are involved in the absorption process, quantum-mechanical selection rules for the transition are different from the one-photon case. So far, two-photon absorption spectra cannot be predicted from one-photon absorption spectra. Importantly, however, emission following one- or two-photon excitation is indistinguishable and occurs from the same quantum-mechanical state. Therefore, in both, the one- and two-photon case, the absorbed energy must be sufficient to lift the electron to the electronic excited state. Consequently, the red spectral edge of absorption for two-photon excitation is twice the wavelength of the one-photon case. The energy between the vibrational ground state of the electronic ground state and the vibrational ground state of the first excited state ${\bar{v}}_{00}$ corresponds to 486 nm (20,565 cm^-1^). With two photons of equal energy this corresponds to a two-photon excitation wavelength for the ${\bar{v}}_{00}$ transition of 972 nm. For voltage imaging ANNINE-6 and derivatives are typically excited at about 510 nm and 1020 nm for one- and two-photon excitation, respectively (Kuhn and Fromherz, 2003; Kuhn et al., 2004) which is both far in the red tail of the absorption spectrum where vibrational energy in the ground state is required to allow a transition.

To study the voltage response of ANNINE-6 with two-photon excitation, linescans were acquired along the membrane of a HEK293 cell while applying external electric fields over the cell (Figure 5C) (Kuhn et al., 2004).

Also in the two-photon case, the signal is a linear function of the applied membrane voltage within the physiological range (Figure 5D,E) (Kuhn et al., 2004). Only if membrane voltages of more than 100 mV are applied, the response becomes slightly non-linear. This non-linearity can be explained by a pure geometric consideration of a linear shift of the excitation and emission spectrum and the resulting response (Kuhn et al., 2004). In other words, the non-linearity is the result of the linear shift of the curved spectrum. As expected, the sensitivity increases near the red spectral edge of absorption (Figure 5D,E) as for one-photon excitation (Kuhn et al., 2004).

For the voltage-sensitive dye di4-ANEPPS (Fluhler et al., 1985) and its derivatives, like JPW1114 (Antic and Zecevic, 1995) or JPW3028 (Antic et al., 1999), the optimal wavelengths for one-photon and two-photon excitation at the red spectral edge are 560 and 1120 nm, respectively. This can be estimated from the two-dimensional spectrum of di4-ANEPBS (same chromophore as di4-ANEPPS) and the corresponding fit (Figures 3, 5 in Kuhn and Fromherz, 2003). Thereby, the sensitivity of 16%/100 mV for two-photon excitation at 1060 nm (Acker and Loew, 2013) might increase to over 20%/100 mV above 1120 nm.

## Solvatochromism

If absorption and emission spectra are solvent-dependent, a dye shows solvatochromism (Reichardt and Welton, 2011). In principle, all charge-shift voltage-sensitive dyes show solvatochromism because the shift of the charge interacts with the solvent shell of the dye, and a polar solvent like water will have a stronger influence on the charge shift than a non-polar solvent like chloroform. To describe solvatochromism, a model is used where the change of the molecular dipole moment upon excitation interacts with the polarizable environment. This model must be refined for hemicyanine dyes used for voltage imaging. In this refined model an electric point charge and an electric point dipole in the center of a sphere interacts with the solvent (Fromherz, 1995). This Fromherz model describes the experimental solvatochromism data of hemicyanine dyes, like RH-160, di4-ANEPPS, and the ANNINE dyes, exceptionally well, by predicting the shift of the excitation and emission spectra in response to a change of the solvent (Fromherz, 1995; Hübener et al., 2003).

In addition to the spectral changes, the quantum yield of fluorescence depends on the solvent (Ephardt and Fromherz, 1991; Reichardt and Welton, 2011). For example, ANNINE-6plus, as a water-soluble derivative of ANNINE-6, is barely fluorescent in aqueous solutions. Therefore, a pipette for dye-loading is barely visible with one-photon or two-photon excitation, but the dye shows bright fluorescence as soon as the chromophore enters the hydrophobic environment of the membrane.

If we now consider a voltage-sensitive dye molecule in a cell membrane and apply an external electric field, then the voltage-sensitive dye might slightly move in or out of the membrane due to the charged head group. If this happens, the excitation and emission spectra and the quantum yield change because of a change in the local solvent shell of the chromophore, i.e., due to solvatochromism. Sometimes this effect increases the spectral change due to the electrochromic shift, but sometimes it decreases it. A voltage-sensing mechanism based on a combination of electrochromism and solvatochromism depends not only on the electric field over the membrane but also on the local environment of the voltage-sensitive dye molecules and their mobility relative to the membrane-water interface, which is based on hydrophilic and hydrophobic interactions. Therefore, the relative fluorescence change of dyes which show a voltage-sensing mechanism based on a combination of electrochromism and solvatochromism is membrane-dependent. In other words, the lipid composition of the membrane will influence the sensitivity. Also, as a voltage-sensing mechanism based on solvatochromism results from movement of dye molecules at the membrane-water interface, it is not as fast as the pure electrochromic mechanism. This can be neglected for neuroscience applications but becomes relevant when imaging in the μs range or faster.

## Characteristics of Annine Dyes

When we tested the spectral changes of voltage-sensitive dyes in a neuronal membrane with two-dimensional spectroscopy we found that ANNINE-5 (chromophore with 5 rings), ANNINE-6, and their derivatives show a pure electrochromic behavior, as can be concluded from the symmetric shift in excitation and emission (Kuhn and Fromherz, 2003; Fromherz et al., 2008). di4-ANEPB showed a spectral change in response to a voltage change that indicates electrochromism with a small influence of solvatochromism; while RH-421 showed electrochromism with a strong influence of solvatochromism (Kuhn and Fromherz, 2003). This can be seen in the asymmetric shift of the excitation and emission spectrum (Figures 3, 5 and Table 1 in Kuhn and Fromherz, 2003). The only explanation is that ANNINE-5, ANNINE-6, and even the water-soluble ANNINE-6plus do not move in or out of the membrane in response to a membrane voltage change. The absence of a solvatochromic effect during voltage imaging indicates that voltage imaging will be independent of intracellular and extracellular solutions and the membrane composition. We confirmed this membrane-independent voltage-sensing behavior in leech neurons for ANNINE-6 and ANNINE-6plus (Kuhn and Fromherz, 2003; Fromherz et al., 2008) and in HEK293 cells for ANNINE-6 (Kuhn et al., 2004). The linearity of the voltage response was shown in HEK293 cells for ANNINE-6 (Kuhn et al., 2004) and in cultured hippocampal neurons for ANNINE-6plus (Pages et al., 2011). We tested ANNINE dyes with excitation at the red spectral edge of absorption on different setups, in different laboratories, and in different tissues and recorded reliable voltage signals. Cells, tissue, and animals we tested include Ctenophora, Lymnaea stagnalis, sea urchin egg, zebra fish brain, rat brain slice, mouse barrel cortex *in vivo* (Kuhn et al., 2008), and several other cell cultures. ANNINE dyes have also been successfully used in cardiomyocytes (Bu et al., 2009), mouse kidney cells (Chambrey et al., 2013), outer hair cells (Ramamoorthy et al., 2013), plant cells (Flickinger et al., 2010; Berghoefer et al., 2012), T-lymphocyte cells (for electroporation experiments with 5 ns temporal resolution) (Frey et al., 2006), and others.

Another reason – in addition to hydrophobicity – for the absence of dye movement during membrane voltage changes might be the rigid structure of the chromophore, consisting of anellated benzene rings (Hübener et al., 2003). This rigid design was originally chosen to avoid photoisomerization at double bonds (Ephardt and Fromherz, 1993) and rotamerism at single bonds (Röcker et al., 1996) in non-anellated charge-shift dyes, which are associated with radiationless transition and triplet state generation. The rigid design might also contribute to an orderly alignment of the ANNINE molecules to the membrane normal. This is important because the dipole moment of the dye molecule, which is oriented parallel to the axis of the molecule, and the electric field over the membrane are vectors and their interaction, i.e., modulation during a voltage change over the membrane, is proportional to their dot product (multiplication of magnitudes when parallel; zero when perpendicular) (Figure 1D).

Interestingly, dye flipping has not been observed so far with ANNINE dyes. This is an important feature and allows for extended imaging periods.

Additionally, so far, no pharmacological side effects were observed in experiments using ANNINE dyes. While some voltage-sensitive dyes like di-4-ANEPPS potentiated GABA action, no such effect was observed for ANNINE-6 and ANNINE-6plus (Mennerick et al., 2010). In another study it was shown that ANNINE-5 is photochemically more stable than RH-421 (Amoroso et al., 2006). It was proposed that this stability arises from the more rigid ring structure than the alternating single-double bonds of RH-421.

## Signal-To-Noise Ratio of Voltage Imaging

**Figure 6:**
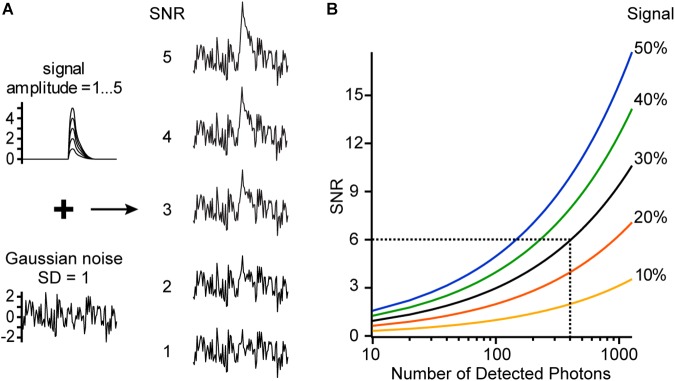
Signal-to-noise ratio (SNR). **(A)** Simulated signal traces with different amplitudes (1–5) and a trace with Gaussian noise (*SD* = 1) are added to generate traces of different SNR. **(B)** SNR is shown as a function of the signal (sensitivity multiplied by membrane voltage change, SΔV [%]) and the number of detected photons. For example, we image with a voltage-sensitive dye which has a sensitivity of 30%/100 mV at a specific excitation wavelength. Now a voltage change of 100 mV occurs in our sample, giving a signal of 30%. At every time point before and after the voltage change, we detect on average 400 photons, resulting in a relative noise level of 5%. Therefore, the measured trace will have a SNR of 6.

Every measurement is composed of the pure signal and noise (Figure 6A). In the case of voltage imaging, the signal (%) is defined by the relative fluorescence change or sensitivity S $\left(\frac{\%}{mV}\right)$ multiplied by the membrane voltage change ΔV (*mV*). Assuming an ideal imaging setup, the noise is caused by fluctuations of the number of detected photons (a non-ideal setup will have additional noise sources like electronic noise or noise due to intensity fluctuations of the excitation light source). Importantly, the incident photon count follows a Poisson distribution. A characteristic of the Poisson distribution is that if the average number of detected photons is n, then the expected fluctuations will be the square root of n. In other words, the standard deviation of *n* is $\sqrt{n}$. Therefore, the relative noise level is $\frac{\sqrt{n}}{n}$ or, in %, $\frac{\sqrt{n}}{n}$100%.

In voltage imaging experiments, it is necessary to optimize the signal-to-noise ratio (SNR) (Kuhn et al., 2004). The SNR is the ratio of the relative signal (in %) divided by the relative noise (in %) (Figure 6B).

$$SNR\;=\;\frac{S\text{Δ}V}{\frac{\sqrt{n}}{n}100\%}\;=\;\frac{S\text{Δ}V\sqrt{n}}{100\%}$$

This formula is crucial because it shows the importance of the relative fluorescence change or sensitivity *S* (Figure 4C). For example, by doubling the sensitivity, the SNR doubles or, alternatively, only a quarter of photons are needed to reach the same SNR. The number of detected photons *n* is proportional to the number of dye molecules available, to their absorption coefficient and quantum efficiency, and to the intensity of the excitation light source. This means that *n* is proportional to the intensity of the fluorescence spectrum (Figure 2, 4A), but the SNR increases only with the square root of *n*.

The equation also shows that, theoretically, any SNR can be reached for any signal SΔV by adapting the number of detected photons *n*. The number of detected photons can be easily estimated. In a first approximation, a well set-up two-photon microscope is shot noise-limited. From a background-corrected time course of a constant fluorescence signal, measured in arbitrary units, the mean intensity and standard deviation of the intensity can be calculated. The mean number of photons (n) can then be estimated from this equation:

$$n\;=\;{\left(\frac{mean}{std}\right)}^{2}$$

Temporal or spatial averaging increases the number of photons taken into account, and therefore reduces the relative noise $\left(\frac{\sqrt{n}}{n}\right)$ accordingly.

## Optimizing Voltage Imaging

For all fluorescence imaging, it is important to keep the number of excited dye molecules low, in order to avoid phototoxicity and bleaching. This is especially true for voltage imaging since the probes are close to indispensable membrane proteins. Excited voltage-sensitive dye molecules can cause photochemical reactions (see for example Schaffer et al., 1994) by generating oxidizing singlet oxygen molecules (Kalyanaraman et al., 1987) or, if non-anellated, undergo excited-state isomerization (Fromherz and Heilemann, 1992; Röcker et al., 1996; Amoroso et al., 2006). Therefore, it is important to excite as few dye molecules as possible and to detect as many emitted photons as possible. At the same time, detected photons should carry as much information about the membrane voltage as possible. Therefore, voltage-sensitive dye molecules should only be excited where the sensitivity is highest, and, at the same time, where all generated fluorescence carries a voltage signal with the same sign (positive or negative) (Figure 4C).

To convert the spectral shift into a detectable intensity change it is necessary to select an excitation band or line, and a corresponding emission band that optimizes the signal. For electrochromic charge-shift probes, there are always two spectral ranges with high sensitivity (in the following all wavelength bands refer to ANNINE-6 and ANNINE-6plus): One is relatively independent of the excitation wavelength (Figure 4C; 360–450 nm), but selective for the emission wavelength range close to the blue spectral edge (Figure 4C; 520–550 nm). This spectral range should not be used for voltage imaging because many dye molecules are excited, but only in a narrow spectral range (at the blue edge of the emission spectrum) a large fractional (%) intensity change will occur during a voltage change. The other spectral range is selective for the excitation wavelength (Figure 4C; 490–510 nm), but relatively independent of the emission wavelength (Figure 4C; 550–750 nm). In this case, all emitted photons originate from a spectral range with high sensitivity, i.e., high information content, with the same sign (in this case, negative relative fluorescence change) and therefore should be selected for detection, using the emission filter.

**Figure 7:**
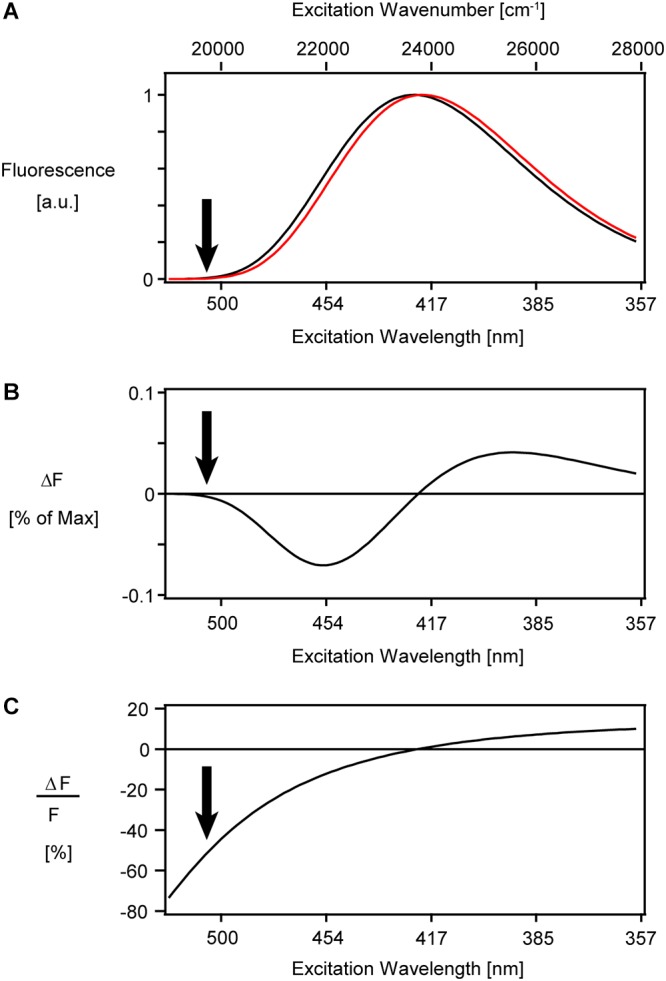
Simulation of a pure shift of the excitation spectrum of ANNINE-6. **(A)** A lognormal function (Siano and Metzler, 1969) resembling the ANNINE-6 excitation spectrum (Kuhn and Fromherz, 2003) was blue shifted by about 3 nm (red), equal to a shift of 160 cm^-1^ in wavenumber. This spectral shift corresponds to a 100 mV membrane voltage change. **(B)** The difference between the two spectra in **(A)** shows extrema at the steepest positive and negative slope. The negative peak amplitude of fluorescence change at 454 nm is only –7% of the peak intensity at 420 nm. **(C)** The simulated relative fluorescence change diverges at the red spectral edge of the excitation spectrum. In reality, the sensitivity has a thermodynamic limit. To image voltage with ANNINE-6 or ANNINE-6plus (which has the same spectral properties), an excitation wavelength of 480 nm or higher is chosen. Arrows indicate a typical excitation wavelength for voltage imaging at 510 nm, corresponding to 1020 nm with two-photon excitation. The absorption coefficient is less than 5% of that at the peak of absorption. The here shown spectra are simulated under the assumption that the full emission spectrum is integrated, and therefore the fluorescence change and relative fluorescence change are independent of the emission wavelength.

In case of excitation at the very red spectral edge of the excitation spectrum and collecting all emitted photons, the sensitivity will be independent of the spectral shift of the emission spectrum. Figure 7A shows a lognormal function resembling the ANNINE-6 excitation spectrum. A pure shift of the spectrum results in a fluorescence change with extrema at the steepest slopes (Figure 7B), and a sensitivity that diverges at the spectral edge (Figure 7C).

With the voltage sensitivity increasing more and more toward the red spectral edge, the question remains: Is there a limit of voltage sensitivity for a given dye? The two-dimensional sensitivity spectrum of Figure 4C implies a higher and higher sensitivity toward the red spectral edge of absorption until it fades in the noise above an excitation wavelength of 500 nm. Thermodynamic considerations show that there is a theoretical limit of voltage sensitivity for a given dye (Kuhn et al., 2004). In short, the spectral edge is expected to follow a Boltzmann distribution and therefore decay exponentially. This decay of the spectral tail considers only one vibrational mode of the chromophore and is therefore the steepest possible decay. In more realistic models, additional vibrational modes must be taken into account which would flatten the spectral tail. If this spectral tail is now shifted due to an external electric field, the steepest spectral tail will give the highest voltage sensitivity. Therefore, this simple model of the spectral Boltzmann tail will give us a theoretical thermodynamic limit for voltage sensitivity for a given dye. For ANNINE-6 this limit is -0.77%/mV for extracellular application of the dye and a positive voltage change of the cell (Kuhn and Fromherz, 2003; Kuhn et al., 2004). The highest measured sensitivity for ANNINE-6 is -0.52%/mV (with two-photon microscopy at an excitation wavelength of 1040 nm, corresponding to 520 nm with one-photon excitation) (Kuhn et al., 2004). For comparison, the theoretical limit of voltage sensitivity for di-4-ANEPBS and RH-421 is 0.43%/mV and 0.24%/mV, respectively (Kuhn and Fromherz, 2003; Kuhn et al., 2004).

Excitation at the red spectral edge of absorption has additional advantages which might be interesting for imaging applications other than voltage imaging: (1) The amount of energy converted into heat during the relaxation process after excitation is minimized. (2) The probability for intersystem crossing is minimized, as the energy levels with high probability of crossing might not be excited any more. Both effects reduce phototoxicity and contribute to an extended imaging time and/or increased SNR.

## Applications of Two-Photon Voltage Imaging With Annine Dyes

**Figure 8:**
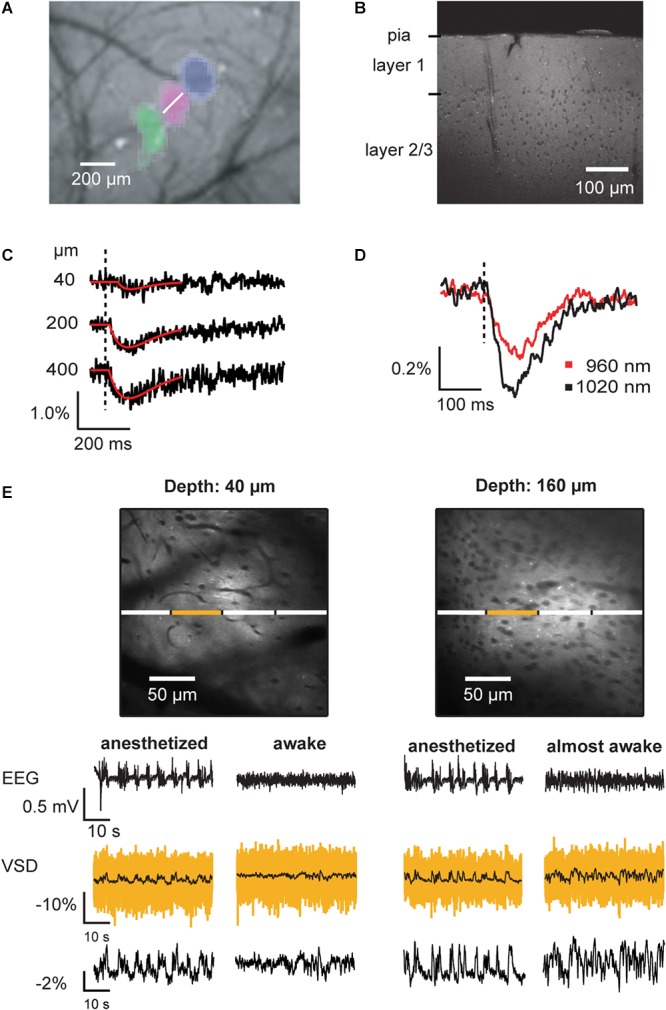
Voltage imaging of average membrane potential of neuronal tissue *in vivo*. **(A)** Imaging of intrinsic signals reveals the location of primary responses in barrel cortex. In this montage, three vibrissa responses (green, magenta, blue) in relation to the blood vessel pattern are shown. Following the imaging of intrinsic signals, the brain region of interest was bulk loaded with VSD. **(B)** Two-photon image of a barrel cortex brain slice prepared after *in vivo* bulk-loading with ANNINE-6 shows homogeneous labeling. Somata (here of layer 2/3) and blood vessels remain dark. **(C)** Two-photon linescans performed at the location indicated in **(A)** (white line) at different depths below the brain surface were spatially averaged. Vibrissa stimulation causes an average membrane depolarization, which results in decrease of fluorescence if the dye is bound to the outer leaflet of the membrane and the dye is excited at the red spectral absorption edge. **(D)** Under the same experimental conditions, the excitation wavelength was changed. As expected from a purely electrochromic dye, excitation closer to the spectral edge results in a larger relative fluorescence change. Such an experiment confirms the mechanism of voltage sensing. **(E)** *In vivo* two-photon images of cerebral cortex layer 1 and layer 2/3. Blood vessels and somata appear as dark shadows, as the dye is bound to the outer leaflet of the cell membranes. Line scans allow measurement of local average membrane depolarization in anesthetized and awake animals, which correlates with the local electro-encephalogram (EEG). The yellow trace is the averaged trace of the line scan segment indicated above. The black, overlaid trace is the corresponding filtered trace (boxcar smoothing, 200 ms). The segmentation allows analyzing cross-correlations among neighboring brain segments. For example, cross-correlations among segments are higher in anesthetized than in awake animals (Kuhn et al., 2008). The trace at the bottom shows the relative fluorescence change of the full linescan after filtering with 200 ms boxcar smoothing. EEG signals are biphasic, representing sources and sinks while the VSD signal shows average membrane depolarization (negative fluorescence change). As the neuronal activity de-correlates during the transition from anesthetized to awake state, the average voltage signal becomes noisy (160 μm below the dura mater, right VSD traces) and then flat (40 μm, right VSD traces) in the fully awake animal. Average membrane voltage measurements can be recorded for hours without photo bleaching. Copyright (2008) National Academy of Sciences, United States (Kuhn et al., 2008).

With the above described mechanistic understanding and optimizations of two-photon voltage imaging with ANNINE dyes, a wide variety of applications are possible. For example, ANNINE-6 can be bolus loaded *in vivo* and the average membrane potential can be measured (Figure 8) (Kuhn et al., 2008). Sensory responses or brain oscillations up to at least 15Hz can be observed.

**Figure 9:**
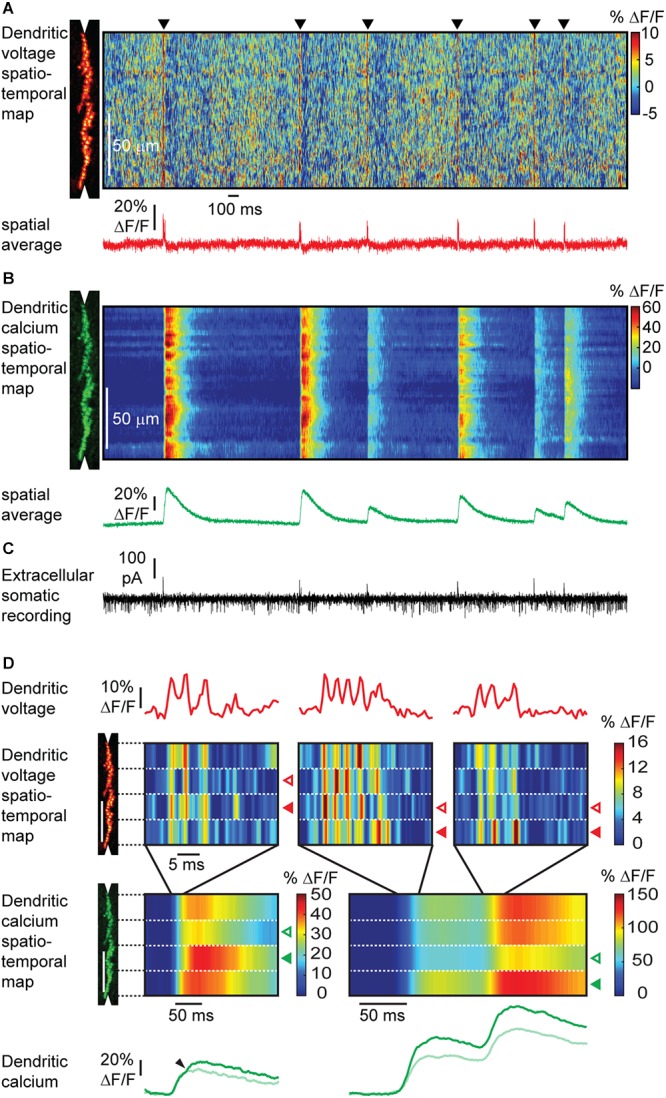
Simultaneous voltage and calcium imaging of Purkinje neuron dendrites and electric recording from the soma in the awake mouse. **(A)** A linescan at 2 kHz was taken along the Purkinje neuron dendrite (left) to measure a voltage spatio-temporal map in an awake mouse. Note red vertical lines (black triangles) indicating activation of the full dendritic tree during a complex spike, and background pattern with red ‘hotspots’ indicating localized subthreshold electrical activity. The spatially averaged dendritic voltage trace shows dendritic complex spikes at high SNR. **(B)** The corresponding dendritic calcium spatio-temporal map shows large transients for every dendritic complex spike. Note the spatial variability of the peak amplitude of calcium transients within single dendritic complex spikes and the amplitude variability between different dendritic complex spikes. **(C)** The access port allowed simultaneous extracellular electrical recordings from the soma while imaging voltage and calcium transients from the dendrites. Simple spikes (somatic Na^+^ spikes) result in a current sink at the soma, while complex spikes (dendritic Ca^2+^ spikes) result in a dominant current source signal at the soma. **(D)** Different parts of the dendritic tree show a different number of spikelets during the same complex spike event. The number of spikelets correlate with the amplitude of the calcium transients in each part of the dendritic tree. Open arrowheads indicate spatially localized low activity, filled arrowheads high activity. Spatially localized dendritic spikelets during complex spikes correlate with a local boost in the dendritic calcium transient (small arrowheads). Note that the depolarization is caused by the influx of Ca^2+^. Therefore, the voltage and calcium signal should have the same onset. The observed delay in onset of the calcium signal is the result of the dynamics of the calcium indicator GCaMP6f (Roome and Kuhn, 2018).

To overcome labeling of all cell surfaces and a resulting mixture of voltage signals, ANNINE-6plus can be internally loaded into single cells *in vivo* (Figure 9) (Roome and Kuhn, 2018). A necessary requirement for this experiment is a chronic cranial window with access port (Roome and Kuhn, 2014) which allows access to the brain to electroporate single neurons, guided by two-photon microscopy. Suprathreshold as well as localized subthreshold events can be detected in dendrites of Purkinje neurons in awake animals with high spatial and temporal resolution. In this case, the labeling after a single electroporation lasts for at least 2 weeks and therefore allows for long-term single neuron imaging experiments. The access port also allows electrical recording, and virus, dye and drug application in combination with the voltage imaging technique. Additionally, voltage imaging with ANNINE dyes can be easily combined with green calcium indicators, specifically GCaMP6 (Roome and Kuhn, 2018). All these experiments can be done without bleaching and with neglectable phototoxicity for many minutes of repetitive laser excitation.

Detailed protocols for these experiments will be available in Multiphoton Microscopy, Springer Nature Neuromethods (Kuhn and Roome, 2019). We hope, that in combination with this paper, it will be possible to adapt the experimental conditions for a wide range of voltage imaging applications on different experimental setups and with different cell types.

## Author Contributions

BK and CJR wrote the manuscript.

## Conflict of Interest Statement

The authors declare that the research was conducted in the absence of any commercial or financial relationships that could be construed as a potential conflict of interest.
